# Circulating MicroRNAs as Potential Biomarkers of Atrial Fibrillation

**DOI:** 10.1155/2017/7804763

**Published:** 2017-03-02

**Authors:** Ananília Medeiros Gomes da Silva, Jéssica Nayara Góes de Araújo, Renata Caroline Costa de Freitas, Vivian Nogueira Silbiger

**Affiliations:** Department of Clinical and Toxicologic Analyses, Federal University of Rio Grande do Norte, Natal, RN, Brazil

## Abstract

Atrial fibrillation (AF) is the most common supraventricular arrhythmia in the population. MicroRNAs (small endogenous noncoding RNAs) are attractive candidates as biomarkers for AF, especially considering that miRNAs are stable and are detected within easily accessible biofluids such as blood. In this review, we selected twelve studies (2012 to 2016) that were classified according to the sample type. We aimed to provide an overview of the role of circulating miRNAs in AF and to discuss the variability of the results, seeking to improve the perspective of the use of miRNAs as potential noninvasive biomarkers for this heart disease.

## 1. Introduction

Atrial fibrillation (AF) is a supraventricular arrhythmia that occurs when electrophysiological abnormalities alter the atrial tissue, promoting formation and/or propagation of abnormal electrical impulse. It is the most common type of arrhythmia in clinical practice, and it is a significant health, social, and economic problem, increasing the risk of stroke, heart failure, and morbidity and disability, especially in the elderly [1]. According to the American Heart Association, AF can be classified as first detected episode of AF, paroxysmal when AF is sustained for less than 7 days and terminates spontaneously (PAF), persistent when AF is sustained beyond 7 days (PersAF, can be long-standing when AF persists for more than 1 year), and permanent when cardioversion attempts have failed or have not been tried [2]. The diagnosis of AF is based on the evaluation of symptoms and intermittent rhythm monitoring; however, those approaches are often inconclusive or unsatisfactory; moreover, most patients are asymptomatic [3]. Therefore, there is a demand for biomarkers of diagnostic and/or prognostic value in AF.

Electrical remodeling and structural remodeling of the atria cause changes that influence the development and maintenance of AF, and they might be associated with genetic modulations mediated by regulatory molecules (e.g., noncoding RNAs) and protein-encoding genes that are involved in atrial physiology [4]. MicroRNAs (miRNA) are small endogenous noncoding RNAs with ~22 nucleotides that play important gene-regulatory roles by base-pairing with target mRNAs at the posttranscriptional level [5–7]. The mechanisms of miRNA-mediated repression of gene expression involve mRNA degradation or blockage of mRNA translation. In addition, multiple miRNAs have arrhythmogenic potential and different miRNAs are involved in different types of atrial fibrillation. However, the role of circulating miRNA as diagnostic biomarkers for AF is not established [8].

The prominent function of miRNAs in the cardiovascular system has provided new perspective on disease mechanisms and has revealed intriguing diagnostic and therapeutic targets for a variety of cardiovascular disorders [9]. Recently, these molecules have drawn great attention as potential noninvasive biomarkers of diseases, such as atrial fibrillation, since they are readily detected and highly stable in biofluids [4, 10–12]. This review aimed to provide an overview of the role of circulating miRNAs in AF and to discuss the diagnostic potential of miRNAs as biomarkers for this heart disease.

## 2. Search Strategy

In this review, searches in the PubMed public database (https://www.ncbi.nlm.nih.gov/pubmed) were performed using the following key words “plasma miRNA atrial fibrillation,” “serum miRNA atrial fibrillation,” “circulating miRNA atrial fibrillation,” “plasma microRNA atrial fibrillation,” “serum microRNA atrial fibrillation,” “circulating microRNA atrial fibrillation,” and “plasma miR atrial fibrillation,” “serum miR atrial fibrillation,” “circulating miR atrial fibrillation.” Twenty-two studies were identified in this search. Of them, four are review articles, and 18 represent original research. Articles in which the circulating miRNAs were not shown (two research papers) or AF patients were not included (two research papers) and also articles published in languages other than English (two research papers in Chinese) were excluded from the analysis. Based on the analyzed data, we performed classification of circulating miRNA species according to the sample choice, which might influence the diversity of potential circulating biomarkers of AF.

## 3. Plasma miRNA Involved in AF

In the first study that evaluated circulating miRNAs in AF, miR-146a, miR-150, miR-19a, and miR-375 were significantly downregulated, but miR-150 demonstrated more pronounced change, suggesting its potential association with AF [10]. Authors carried out a discovery stage firstly to determine the candidate miRNAs for further investigation in plasma. They used a massively parallel signature sequencing to analyze the miRNA expression profile (miRNome) in plasma of 5 patients with PAF and 5 PersAF, compared to 5 healthy individuals. 243 miRNAs were detected in the PAF group, and 256 miRNAs were detected in the PersAF group. Five specific miRNAs stand out (miR-125a-5p, miR-19a, miR-221, miR-342-3p, and miR-409-3p) and were upregulated in patients with PAF but not in patients with PersAF. Moreover, ten specific miRNAs (miR-146a, miR-589, miR-146b-5p, miR-100, miR-150, miR-199a-5p, miR-199b-5p, miR-375, miR-99b, and miR-320b) were dysregulated between PAF and controls, as well as eleven (miR-146a, miR-148b, miR-19a, miR-221, miR-598, miR-941, miR-100, miR-150, miR-320b, and miR-375) between PersAF and controls. However, only four candidate microRNAs (miRNA-146a, miRNA-150, miRNA-19a, and miRNA-375) met their selection criteria to be evaluated by qRT-PCR in an independent cohort of 90 plasma samples (30 healthy individuals, 30 patients with PAF, and 30 PersAF). Authors reported that samples were processed within 20 minutes of collection using two-step centrifugation, but they did not inform centrifugation conditions. Total RNA was isolated using mirVana PARIS RNA isolation kit (Ambion).

In 2013, Dawson and colleagues demonstrated that plasma miR-29b and miR-21 expression was significantly decreased in patients with AF or congestive heart failure (CHF) in comparison with controls, but the levels of miR-29b were even more decreased in patients with both AF and CHF. CHF causes fibrotic atrial remodeling and contributes to the maintenance of AF. Therefore, miR-29b and miR-21 could be important biomarkers for atrial remodeling. In this investigation, plasma levels of candidate miRNAs were determined by qRT-PCR, and the study group consisted of AF 17 patients without CHF, 32 CHF patients without AF, 16 patients with both AF and CHF, and 30 controls. Authors specified that samples were processed within 4 hours of collection. Plasma samples were obtained after centrifugation at 4000 rpm for 20 min at room temperature, and total RNA was isolated using the mirVana PARIS RNA isolation kit (Ambion) [13].

In the same year, Nishi and collaborators suggested that miR-21 might be involved in AF occurrence based on its significant high expression in human atrial tissue of patients with AF when compared to patients with sinus rhythm. They also investigated miR-21 plasma levels in 16 AF patients compared to 4 healthy volunteers by qRT-PCR, aiming to determine if they would obtain the same pattern of differential expression. Surprisingly, the miRNA-21 plasma levels in patients with AF were significantly decreased, revealing an inverse relationship between the level of miRNA-21 expression in atrial tissue and plasma level. In this study, there is no indication of the approach adopted to prepare plasma samples for RNA isolation, but they mention the utilization of mirVana PARIS RNA isolation kit (Ambion) [14].

Lu and colleagues investigated the changes in expression profile of plasma miRNAs and the regulatory effect of miRNAs associated with AF on ion channels. The study included 112 patients with AF and 112 non-AF as controls, and miRNA expression profiles from plasma mixed pools were analyzed using microarray chips (miRCURY LNA chip). 15 miRNAs were significantly differentially expressed in AF patients compared to controls. The expressions of miR-328, miR-145, miR-222, miR-1, miR-162, miR-432, and miR-493b were downregulated and miR-634, miR-664, miR-9, miR-152, miR-19, miR-454, miR-146, and miR-374a were upregulated. Samples were processed after 30 minutes through centrifugation at 3000 rpm for 10 min, and total RNA was extracted from the plasma mixed pools using mirVana PARIS RNA isolation kit (Ambion) [15].

A study evaluating local and systemic levels of plasma miRNAs and their relation with the presence of AF and left atrial substrate properties revealed a potential important role of miR-328 in the process of atrial remodeling in patients with AF. Blood was collected from the pulmonary vein (PV) and the left atrial appendage (LAA) of 30 patients with AF, 20 with paroxysmal AF and 10 with persistent AF, undergoing PV isolation, and 10 control subjects with Wolff-Parkinson-White syndrome. Plasma levels of miR-1, miR-26, miR-133a, miR-328, and miR-590 were determined by qRT-PCR. This study indicates that local production of miR-328 in the left atrium might be involved in the process of atrial remodeling in patients with AF, since plasma levels of miR-328 were higher in patients with AF than in controls, and they were significantly higher in the left atrial appendage than in the periphery and PV in patients with AF. In addition, plasma miR-1 levels were also higher in the LAA than in the PV in AF patients. Authors did not inform conditions of sample processing, but they stated that total RNA was extracted using TRIzol LS RNA Isolation Kit (Invitrogen) [1].

Circulating miR-21 and miR-150 were associated with AF in a study that included 112 patients with AF compared to 99 individuals with no AF. This study evaluated plasma levels of 86 miRNAs implicated in the pathogenesis of atrial remodeling or AF, which were selected in a public database, by qRT-PCR using the BioMark System. Plasma miR-21 and miR-150 were significantly lower among patients with AF than in those without AF. Although authors did not inform their conduct for plasma processing, they specified that plasma samples were thawed and centrifuged at 8,000 ×g for 5 minutes and RNA was isolated using the miRCURY RNA Isolation Kit [16].

Another study that aimed to reveal circulating miRNAs as biomarkers associated with AF included 88 patients with PAF, 8 with PersAF and 4 with long-standing persistent AF, and 100 healthy individuals. This investigation examined the levels of AF-specific miRNAs in plasma of patients after catheter ablation using Solexa sequencing, and it showed a total of 389 and 517 miRNAs detected in the AF group and the control group, respectively. Five miRNAs (miR-454, miR-374a, miR-9, miR-152, and miR-664) were found to be upregulated in the AF group, while 11 miRNAs (miR-874, miR-486-5p, miR-328, miR-338-5p, miR-766, miR-409-3p, miR-16-2, miR-487b, miR-493, miR-432, and miR-4732-3p) were downregulated. These 16 candidate miRNAs were also evaluated by qRT-PCR in two sample pool pairs, revealing that miR-432, miR-409-3p, and miR-328 were downregulated in the AF group, which was similar to the sequencing results. The expressions of the other 13 miRNAs were inconsistent with the sequencing results or the dynamic trends were obscure. Blood samples were processed within 1 h of collection through centrifugation at 2,000 ×g for 10 min at 4°C, and total RNA was isolated from plasma using miRNA extraction kit from BioTeke [17].

Recently, a study identified a SNP in the 3′UTR of the SHOX2 gene that has been shown to create a novel miRNA target site for miR-92b-5p. This gene encodes a transcription factor that was previously suggested as a possible susceptibility gene for cardiac arrhythmias, and the authors suggested that the expression of miR-92b-5p in individuals carrying the 3′UTR variant might have a proarrhythmogenic effect, leading to atrial fibrillation. In order to investigate whether the circulating miR-92b-5p levels reflected this condition, the authors evaluated plasma levels of miR-92b-5p in 23 AF patients and 12 patients with sinus rhythm by qRT-PCR. They did not find significant differences between these groups; however, they showed that patients with AF carrying the 3′UTR variant had significantly decreased miR-92b-5p plasma levels compared to AF patients that did not have this polymorphism. In addition, the authors describe that plasma samples were obtained within 4 h of collection after a centrifugation at 4000 rpm for 20 min at RT and RNA was isolated using miRNeasy kit (Qiagen) [18].

## 4. Serum miRNA Involved in AF

One study investigated the association of miR-126 in serum of patients with heart failure (HF) and AF using qRT-PCR analysis. The study group consisted of AF patients (AF group, *n* = 35), HF patients (HF group, *n* = 32), and patients with both HF and AF (HF-AF group, *n* = 36) that were compared to the control group (*n* = 32). miR-126 was downregulated in the 3 patient groups when compared with controls, but it was significantly lower in HF-AF group than in the other groups. Considering that AF may be related to the severity of HF and serum miR-126 expression levels in patients with AF, HF, and HF-AF are low, especially in those with HF-AF, authors suggested that miRNA could serve as a potential candidate biomarker in evaluating the severity of AF and HF. Serum samples were obtained after centrifugation at 3,000 ×g for 10 min at room temperature and total RNA was isolated using miRNeasy kit (Qiagen) [19].

Patients undergoing coronary artery bypass grafting can develop AF, featuring a postoperative atrial fibrillation (POAF). Harling and colleagues evaluated the potential of circulating miRNAs as preoperative biomarkers for AF. They assessed miRNAs expression through microarray, and sixteen miRNAs were differentially expressed in the 11 atrial myocardia of POAF patients when compared with those maintaining sinus rhythm (*n* = 11). Selected candidate miRNAs (miR-483-5p and miR-208a) were subsequently validated individually by qRT-PCR in serum of 13 patients with AF and 21 without AF at different time points: preoperatively, 48 hours postoperatively, and 96 hours postoperatively. miR-208a was not detected in any time point and miR-483-5p was significantly higher preoperatively but not at 48 h or 96 h. Thus, validation of circulating miR-483-5p is necessary to determine the reliability of this biomarker for evaluation of POAF. Authors described that serum samples were obtained after centrifugation at 5,000 ×g for 6 min at room temperature; however, they did not inform the method of RNA isolation [20].

## 5. Platelets miRNA Involved in AF

miRNAs have been shown to participate in platelet function, vascular homeostasis, and inflammation. In addition, levels of platelet miRNAs in the circulation are associated with the risk for cardiovascular diseases, suggesting that platelet-derived miRNAs might have important roles as biomarkers of cardiovascular disease susceptibility, prognosis, or treatment [7]. An in silico study suggested that differential expression of miRNAs in platelets and their target mRNAs in peripheral blood cells might be associated with variability in platelet reactivity, drug response, and drug-induced toxicity [21].

Human platelets contain miRNAs and miRNA processing machinery, but their contribution to platelet function remains incompletely understood [22]. These miRNAs regulate mRNA translation inside the cell and can be delivered to endothelial cells, affecting their function. A study evaluated platelet-derived miRNAs levels by qRT-PCR in 41 patients with HF, 15 with both HF and AF and 26 without AF, and 35 controls. miR-150 levels were 3.2-fold lower in platelets of patients with HF-AF relative to those without AF. This was also observed in serum samples from the same patients, in which miR-150 levels were 1.5-fold lower in patients with HF-AF. Therefore, miR-150 expression levels in platelets of patients with systolic HF and AF are significantly reduced and correlated to the cell-free circulating levels of this miRNA. Serum samples were obtained 2 to 9.5 h after collection, and total RNA was extracted using mirVana PARIS RNA isolation kit (Ambion) [23].

## 6. Whole Blood miRNA Involved in AF

Another study associated miRNA miR-328 with AF occurrence. Its expression was significantly lower among patients with prevalent AF compared to individuals with no AF. In this study, a miRNA expression profiling of 385 miRNAs was performed in 153 AF patients and 2185 individuals with no AF using a high-throughput qRT-PCR platform, the BioMark dynamic array. However, only miR-328 remained significantly associated with prevalent AF after adjustment for risk factors, RNA quality, and concentration. The authors informed that RNA isolation was performed in whole blood samples using the PAXgene Blood RNA Kit [11].

## 7. Final Considerations

The study of miRNAs in biofluids such as blood has expanded in the last few years due to their stability in this specimen and wide-ranging biological potential, becoming attractive candidates as noninvasive biomarkers for a variety of disease processes, including AF [24]. From the twelve studies selected for this review, eight were in plasma, two were in serum, only one was in serum and platelets, and another one was in whole blood. Interestingly, we did not find studies that addressed circulating exosomes, which are important extracellular vesicles filled with RNAs, microRNAs, and proteins and crucial for intercellular communication [25].

In this review, we observed a diversity of miRNAs described as associated with AF and poor overlap of results among different studies. The variability of miRNAs observed in all studies could be related to the different types of samples (plasma, serum, platelet, and whole blood) and methods of analysis used, and the intrinsic diversity of each population included in the studies could also have been influencing the results. Among the 17 miRNAs suggested as good candidates for biomarkers of AF (Table 1), only three miRNAs (miR-21, miR-150, and miR-328) were found to be downregulated in more than one study, suggesting that they might have an important role in AF development. Intriguingly, miR-21 was the only one that had significantly decreased expression levels in three different studies using plasma and these studies coincidentally observed this expression profile in patients with PersAF [13, 14, 16]. Therefore, miR-21 might serve as a strong biomarker candidate for this type of AF.

There has been much discussion around which is the best specimen type for circulating miRNA analysis. Despite divergence on the sample of choice, the idea that methodological variations in sample processing and measurement of extracellular miRNA have substantial influence over miRNAs levels detected seems unanimous [26, 27]. However, some of the recent reports of circulating miRNA profiles in AF lacked details of sample processing and quality control, making it difficult to evaluate whether the results reflect the biological state of samples. It is currently recommended that cell-free miRNAs be obtained from plasma samples after a two-step centrifugation, in which the first centrifugation is important to collect plasma free of circulating cells, while the second can remove residual platelets [28]. The second centrifugation is not necessary for serum due to the coagulation of the sample [24]. However, only one study reviewed here used this kind of processing [10]. As shown in Table 2, there is an important variability in conditions of sample processing and methods of RNA isolation among studies reviewed here. These preanalytical and analytical factors might affect the amount of miRNA present in a given sample. Therefore, there is a need for development of standardized protocols of sample processing and analyzing miRNA levels in biofluids, which will increase the reliability and reproducibility of the results.

Regarding the description of the study groups and clinical parameters, none of the studies described whether patients were in AF at the moment of blood sampling (new-onset AF), which would be important information to be discussed, since it is possible that a patient in new-onset AF has different expression profile in comparison with patients that have AF but were in sinus rhythm during blood collection. Patients in AF have more significant electrical changes that would cause electrical remodeling of the atrium; therefore these patients probably have different patterns of miRNA expression.

Another important factor observed is the prevalence of studies that address AF associated with HF, demonstrating that HF is a major predisposing factor or consequence for the maintenance and progression of AF. This strong association is justified by the significant role of the left atrium in maintaining cardiovascular homeostasis in heart failure, causing structural and electrical changes, implying the development of AF [29].

Moreover, the diversity of the miRNA associated with AF could be explained by the sample size, which varied from 13 to 153 patients [11, 20]. This is an important point for the determination of a potential biomarker, since it is possible that, in small sample groups, less abundant miRNAs are hidden, especially depending on the statistical approach used to analyze the results. However, it is also crucial to observe that in many cases obtaining samples is difficult. Careful screening and recruitment of patients are time-consuming, which might be a limitation leading to small sample sizes. Therefore, the evaluation of circulating miRNAs levels in AF with larger sample sizes might produce more reliable and compatible results.

In conclusion, future research will be necessary to determine reliable circulating miRNAs as biomarkers for early diagnosing, monitoring, and treatment of AF, using similar and detailed methodological standards to avoid the variability of miRNA detected among studies. This could be achieved through international consortia among several research groups working with AF. The efforts to accurate optimization of protocols for miRNA assessment will help to improve the reliability and reproducibility of the results, which are important when considering the clinical use of these molecules.

## Figures and Tables

**Table 1:** Principal circulating miRNAs involved with atrial fibrillation.

Authors	Casuistic	Upregulated	Downregulated
Liu et al., 2012			miR-19a
	30 PAF and 30 PersAF		miR-146a
	30 no AF		miR-150
			miR-375

Dawson et al., 2013	16 AF with CHF and 17 AF without CHF		miR-21
	32 no AF with CHF and 30 controls		miR-29b

Nishi et al., 2013	16 AF		miR-21
	4 no AF		

McManus et al., 2014	2185 no AF		miR-328
	153 AF (prevalent )		

Goren et al., 2014	15 AF with HF	miR-150
	26 HF without AF and 35 controls	

Soeki et al., 2016	20 PAF and 10 PersAF (undergoing PV isolation)	miR-1	
	10 Wolff-Parkinson-White syndrome (no AF)	miR-328	

Wei et al., 2015	35 AF and 36 AF with HF		miR-126
	32 HF without AF and 32 controls		

McManus et al., 2015	112 AF		miR-21
	99 no AF	miR-150

Lu et al., 2015		miR-9	miR-1 miR-145 miR-162 miR-222 miR-328 miR-432 miR-493b
		miR-19	
		miR-146	
	112 AF	miR-152	
	112 no AF	miR-374a	
		miR-454	
		miR-634	
		miR-664	

Hoffmann et al., 2016	17 AF T/T genotype		miR-92b-5p (AF carrying the SHOX2
	6 AF T/C genotype		3′UTR c.*∗*28C allele)

Harling et al., 2017	13 AF	miR-483-5p	
	21 no AF		

Liu et al., 2016	88 PAF, 8 PersAF, and 4 long-standing PersAF		miR-409-3p
			miR-432
	100 no AF		miR-328

PAF, paroxysmal AF; PersAF, persistent AF; CHF, congestive heart failure; PV, pulmonary vein.

**Table 2:** A review of sample preparation protocols and methods of miRNA detection in circulating miRNA.

Authors	Sample type	Sample processing conditions	Method of RNA isolation	Method of miRNA detection
Hoffmann et al., 2016	Plasma	<4 h of collection; centrifugation 4000 rpm, 20 min RT	miRNeasy kit (Qiagen)	qRT-PCR
Nishi et al., 2013	Plasma	Not described	mirVana™ PARIS RNA isolation kit (Ambion)	qRT-PCR
McManus et al., 2014	Whole blood	Not described	PAXgene Blood RNA Kit (PAXgene)	qRT-PCR, BioMark dynamic array
McManus et al., 2015	Plasma	Centrifugation 8,000 ×g, 5 min	miRCURY RNA Isolation Kit (Exiqon)	qRT-PCR
Dawson et al., 2013	Plasma	<4 h of collection; centrifugation 4000 rpm, 20 min RT	mirVana PARIS RNA isolation kit (Ambion)	qRT-PCR
Goren et al., 2014	Platelets and serum	Platelets: stand for 2 to 9.5 h of collection; centrifugation 1,800 ×g, 30 min RT; serum: stand for 2 to 9.5 h	mirVana PARIS RNA isolation kit (Ambion)	qRT-PCR
Wei et al., 2015	Serum	Centrifugation 3,000 ×g, 10 min RT	miRNeasy kit (Qiagen)	qRT-PCR
Harling et al., 2017	Serum	Centrifugation 5,000 ×g, 6 min at RT	Not described	qRT-PCR
Liu et al., 2012	Plasma	<20 min of collection; two-step centrifugation (not described)	mirVana PARIS RNA isolation kit (Ambion)	MPSS and qRT-PCR
Liu et al., 2016	Plasma	<1 h of collection; centrifugation 2,000 ×g, 10 min 4°C	miRNA extraction kit (BioTeke)	Solexa sequencing and qRT-PCR
Lu et al., 2015	Plasma	Centrifugation 3000 rpm, 10 min	mirVana PARIS RNA isolation kit (Ambion)	miRCURY™ LNA chip
Soeki et al., 2016	Plasma	Not described	TRIzol LS RNA Isolation Kit (Invitrogen)	qRT-PCR

RT, room temperature; qRT-PCR, quantitative real-time polymerase chain reaction; MPSS, massively parallel signature sequencing.
